# Pectin Stabilized Fish Gelatin Emulsions: Physical Stability, Rheological, and Interaction Properties

**DOI:** 10.3389/fnut.2022.961875

**Published:** 2022-07-13

**Authors:** Sheng Huang, Hui Wang, Shu Wang, Xiaomei Sha, Ning Chen, Yueming Hu, Zongcai Tu

**Affiliations:** ^1^State Key Laboratory of Food Science and Technology, Nanchang University, Nanchang, China; ^2^National R&D Center for Freshwater Fish Processing, Jiangxi Normal University, Nanchang, China; ^3^Engineering Research Center for Freshwater Fish High-Value Utilization of Jiangxi, Jiangxi Normal University, Nanchang, China

**Keywords:** pectin, fish gelatin, emulsions, non-covalent interactions, interface performance, rheological properties

## Abstract

Pectin, a kind of natural polysaccharide, shows the attractive potential as a natural stabilizer for protein emulsion. The aim of this study is to investigate the effect of pectin on the physical stability, rheology, interface, and interaction properties of the fish gelatin (FG) emulsion, as pectin was utilized to improve the stability of FG, fish oil emulsion. During the study, when pH < 6, the FG-pectin emulsion displayed better storage stability and salinity tolerance. Analyzing the result, pectin could avoid phase separation at the freeze-thaw process and prevent the liquid-gel transition of FG emulsions during storage. On the other hand, when pH ≥ 6, the emulsion displayed high viscosity due to the complex flocculation and stratified during long-term storage. Electrostatic interactions, hydrophobic interactions, and hydrogen bonding of the FG-pectin complexes in the emulsion were all reduced. Overall, pectin improved the stability of FG emulsions through electrostatic repulsion, hydrophobic interactions, and steric hindrance.

## Introduction

Pectin is an anionic polysaccharide derived from plant cell walls and intercellular extracted from orange peel, apple peel, or jujube (1, 2). It is a dietary fiber, which is conducive to weight control and intestinal health, universally utilized as a food additive or medicine carrier material (3). Gelatin is the most commonly used emulsifier, gel, and food supplement needed for upholding the tissues of the human body (4). Based on material sources, it can be divided into mammalian gelatin (e.g., pig and cow), poultry gelatin, and fish gelatin (FG) among others (5). Among them, FG is extracted from various fish byproducts (skin/scale/bone), which have a cheap raw material (fish scale) and low risk of zoonotic diseases (6).

Many studies have focused on the interaction and application of pectin and gelatin. They can form a complex through complex coacervation, edible film, or emulsion to enhance performance on both sides as drug or nutrient delivery systems (7–9). The entrapment and transportation of functional oils by biomacromolecule emulsion are a hot topic in the nutrient delivery process (10). Fish oil has been widely studied because it is rich in docosahexaenoic acid (DHA, C22:6n3) and eicosapentaenoic acid (EPA, C20:5n3), which can effectively reduce plasma triglycerides (11). FG-pectin-embedded fish oil is more conducive to health production and application. But the emulsifying performance of pectin is not ideal; its emulsifying ability is limited by its composition (degree of methyl esterification, protein residues, neutral sugar side chain, and molecular weight), and pectin as an emulsifier is sensitive to environmental pH and ionic strength (12). Although the foaming and water absorption properties of FG are better than those of mammalian gelatin, the emulsifying properties of FG are worse than those of mammalian gelatin (13). Moreover, FG is a linear protein, and its emulsification stability is not as good as that of spherical protein similar to whey protein. Therefore, the application of pectin or FG as a natural emulsifier in production and processing is still limited. In addition, the binding of FG-pectin, similar to most polysaccharide–protein molecules, produces phase separation, which is closely related to their concentrations and other factors (14).

Studies have shown that plant polysaccharides can improve protein emulsion stability (15). For example, gum Arabic enhances the emulsion stability of whey protein isolate (16). Porcine gelatin–pectin-mixed solution was incubated for 4 h under alkaline conditions for conjugation to improve emulsion stability, however, the non-heat-treated gelatin–pectin emulsion was layered after storage for 1 day (8). Besides that, few studies have focused on interfacial rheological changes and interaction forces in the formation of the FG-pectin-stabilized emulsion process. Therefore, the purpose of this study was to improve the storage stability of FG emulsion *via* non-covalent effect and investigate the effect of pectin on the interfacial rheological changes and interaction forces of FG emulsion. The effects of different concentrations of pectin, environmental factors (freeze-thaw, heating, and pH changes), and long-term storage on emulsion stability were investigated, and the underlying mechanisms were analyzed.

## Materials and Methods

### Materials

Pectin (from citrus peel, GalA ≥ 74%, methoxylation degree 54%) was bought from Sigma Co., Ltd. (Shanghai, China). FG (*Tilapia mossambica*, type A, 260 Bloom) was obtained from Ding Biological Technology Co., Ltd. (Suzhou, China). Deep-sea fish oils (DHA + EPA ≥ 80%) were purchased from Guanchen Biological Technology Co., Ltd. (Shanghai, China). All other chemicals were of analytical grade and obtained from commercial sources.

### Preparation of Fish Oil-Loaded Pectin-Fish Gelatin-Stabilized Emulsions

Fish gelatin (2% w/v) and pectin (2%, 3% w/v) were heated to 60°C with continuous stirring to dissolve thoroughly. The emulsions were prepared by “layer-by-layer” approach (Supplementary Figure 1). The 4 mL fish oil was added into the 20 mL FG solution and mechanically sheared by a T18 homogenizer with a 15-mm head at speed of 12,000 rpm for 60 s. Then, 20 mL pectin was added to the previous solution and sheared at the same rate for 30 s. The controlled group was added 20 mL of deionized water instead. To detect the effect of pH on the emulsions, three groups (FG 1.0% w/v and pectin 1.0% w/v, pH 4.2) were adjusted pH values at 6, 8, and 10 with 0.1 M NaOH. To compare the mix method with the layer-by-layer method, 1.0% (w/v) pectin and 1.0% (w/v) FG were mixed before adding fish oil, then following the above process. All the primary emulsions were added into a high-pressure homogenizer (UH-06, Union-Biotech, Shanghai, China) at 100 Mpa pressure of 2 min. Therefore, the final FG concentration of emulsions was 1.0% (w/v) and the aqueous solution to fish oil ratio was 10:1. The fresh emulsion was centrifuged in a high-speed refrigerated centrifuge (10,000 × *g*, 1 h, 4°C) to observe the stratification.

Different concentrations of pectin (0.5%, 1.0%, 1.5% w/v) in emulsions named 0.5%, 1.0%, 1.5%. The emulsion-added deionized water instead of pectin was set as a control group named FE. The various pH groups were pH6, pH8, and pH10. After storage at 25°C a day, the pH6 and pH10 groups were adjusted to pH 4.2 with 0.1 M HCl (referred to as pH6-A and pH10-A, respectively).

### Particle Size and Zeta Potential

Particle size measurements were conducted by the Malvern laser particle size analyzer (Ms3000, Malvern, United Kingdom). The refractive index of the emulsion particles and the aqueous dispersion medium were 1.46 and 1.33 (17). The emulsions were dripped into the test container (diluted by deionized water) until the sample concentration reached the detection limit.

Zeta potential measurements were performed by the Malvern laser particle size analyzer (Nano ZSP, Malvern, United Kingdom). The emulsions were diluted 100-fold with deionized water.

### Emulsion Stability Analysis

The dynamic physical stability (DPS) was determined with a LUMi-Sizer (L.U.M. GmbH, Berlin, Germany) according to the previous report (18). The machine detects emulsions stability *via* horizontal centrifugal force. The centrifugation speed was set at 4,000 rpm for 30 min and the temperature was constant at 25°C.

Freeze-thaw stability test: Froze 5 mL emulsion sample at −20°C for 22 h and then thaw at 30°C for 2 h. Repeat this Freeze-thaw cycle four times to determine DPS, particle size, and zeta potential.

Heat treatment stability test: Heat 5 mL emulsion sample to 60 and 70°C for 30 min in a water bath. DPS, particle size, and potential are then measured after cooling to room temperature.

Influence of salt concentration: Mix 5 mL emulsion sample with NaCl solution, the final NaCl concentrations are 0, 300, 600 mmol/L and total volume is 10 mL, then measure the particle size and potential.

### Storage Measurement

Macroscopic observations: The 3.5 mL of emulsions containing 0.01% (w/v) sodium azide were transferred to a glass container and reserved at 25°C 50% RH. The stratification of the emulsion was observed during the storage.

Optical microscope observations: during the storage, 4 μL emulsion was dropped on a glass slide and then covered by a coverslip. The samples were observed using an inverted microscope (Olympus Co., Ltd., Tokyo, Japan) with a built-in camera with 40-fold objective lens.

### Confocal Laser Scanning Microscopy

The microstructure of emulsions was analyzed by Confocal Laser Scanning Microscopy (CLSM) (Leica TCS SP8 SMD, Germany) according to the method of Miao et al. (19). The emulsion (1 mL) was mixed with 40 μL dye (0.1% Nile red dye and 1.0% Nile blue) and protected from light. Then, 4 μL of samples were added onto a glass slide to observe. Two lasers (488 and 633 nm) were used to excite Nile red and Nile blue, respectively.

### Surface Hydrophobicity

Emulsion surface hydrophobicity was determined by the 8-anilino-1-naphthalenesulfonic acid fluorescent probe (ANS) method according to the method of Li et al. (20) with some modifications. The emulsion (0.5 mL) was mixed with 25 μL 8 mM ANS and 4.5 mL of deionized water. The mix stood in the dark at room temperature for 1 h then measured at λex = 370 nm and λem = 400–600 nm.

### Rheology Properties

The rheological properties of emulsions were determined based on the procedure using the Paar-Physica MCR 302 rheometer (Anton Paar, Ostfildern, Germany) described by Xu et al. (21) with some modifications.

Dynamic frequency sweep: The emulsion (about 17 mL) was poured into a concentric barrel and kept at 25°C for 5 min. The frequency was ranging from 0.1 to 100 rad/s, and the strain value was fixed at 0.5%.

Flow sweep test: The shear rate was increased from 1 1/s to 100 1/s with a fixed strain of 0.5%. The apparent viscosity (AV, η, Pa⋅s) was recorded. AV was modeled as:


(1) $$\eta =D\,\gamma^{n-1},$$

Where D is the consistency index (Pa⋅s*^n^*), γ is the shear rate (1/s), and n is the flow behavior index.

### Interfacial Properties

An optical contact angle measuring instrument (Optical contact angle system OCA, Germany) was used to detect the change in the interface pressure at the oil-water interface. Before the test, the density of water, pectin, FG, and FG-pectin were detected by flat-bottomed spherical density bottles after being treated with T18 homogenizer at speed of 12,000 rpm for 60 s. The 1.0% (w/v) pectin and 1.0% (w/v) FG were mixed and homogenized at 100 Mpa pressure for 2 min, which is named HP 1.0%. Using a syringe with a straight needle, the sample is drawn and got rid of disturbance of bubbles in the liquid. Then, the needle was immersed in a transparent glass tank filled with fish oil. A droplet of 20 μL was applied to analyze the droplet shape in an adsorption time (at least 180 min).

### Interaction Force Analysis

Surface hydrophobicity analysis was measured by the method of Li et al. (20) with some modifications. The method was to add 0.5 mL emulsion and 25 μL of 8 mM ANS to 4.5 mL of deionized water, wait reaction performed at room temperature for 1 h in the dark, then analyzed with a fluorescence spectrometer in λ_*ex*_ = 370 nm and λ_*em*_ = 400–600 nm.

The emulsion (1 mL) was poured into EP tubes, after being freeze-dried, and was subjected to infrared spectroscopic analysis with 32 scans from 500 to 4,000 cm^–1^, according to the method described by Ren et al. (22).

### Statistical Analysis

Three or more replicates were tested independent experiments, and the results were expressed as mean values ± standard deviations. Data analyses were subjected to one-way ANOVA, with the significance level set at *p* < 0.05. The SPSS 22.0 software (SPSS Inc., Chicago, IL, United States) and Origin Pro 9.0 software were used for statistical and graphical analysis.

## Results and Discussion

### Effect of Preparation Method on the Properties of Emulsions

**FIGURE 1:**
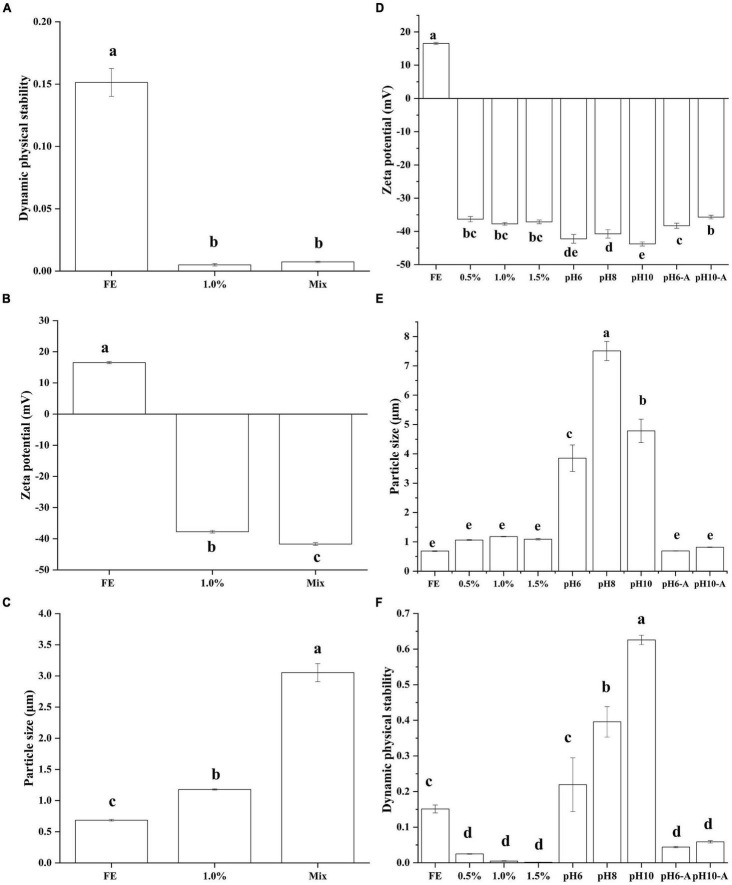
Effect of preparation method on the dynamic physical stability **(A)**, zeta potential **(B)**, particle size **(C)** of complex emulsions; effect of pectin and pH on the zeta potential **(D)**, particle size **(E)**, and dynamic physical stability **(F)** of complex emulsions.

Generally, emulsions stabilized by polysaccharide–protein complexes are fabricated by two alternative methods (23). (1) For mixing before the emulsification method, for example, soy polysaccharide and soy protein mixture are utilized to fabricate soybean oil emulsion (24). (2) For the layer-by-layer method, shear and homogenization are used to form a primary emulsion with gelatin–oil; the primary emulsion is then mixed with pectin to prepare two-layer emulsions. In this study, DPS was used to affect the stability of emulsions (Figure 1A). After centrifugation, different mass phases were divided, resulting in variations in light transmission. These variations were detected by near-infrared spectroscopy. The worse the stability of the emulsion, the greater the variation in transmittance with centrifugal time and the larger the instability index. FE emulsion had a high DPS, which means the disability of pure FG emulsion. Group 1% and Mix had similar DPS (*p* > 0.05) which were lower than FE (*p* < 0.05), indicating that the complex emulsion fabricated by the two methods had good stability. Although some research reported that the layer-by-layer method had a primary problem, its emulsion was prone to bridging or depletion in the flocculation. As such, the mixed emulsion method has better stability than the layer-by-layer method (25). On the other hand, researchers commonly prepare stable emulsion with proteins and polysaccharides through the layer-by-layer method (26). The difference in emulsion stability prepared by the two methods was not obvious in this study.

To further study the difference between the two methods, the difference among FG emulsion, group 1% (layer-by-layer method), and Mix (mixing before emulsification method) in zeta potential and particle size (D_43_) were compared (Figures 1B,C). Zeta potential and particle size are considerable indicators to evaluate the stability of an emulsion system. Pectin brought abundant negative charge from -COO^–^ leading to the negative of the complex emulsion zeta potential. The zeta potential of group Mix was lower than group 1% (*p* < 0.05). The particle size is closely related to the surface properties and dispersing ability of the material. Generally speaking, a smaller particle size emulsion has better spatial stability (27). Pectin complex emulsion had a higher particle size than FG emulsion (*p* < 0.05). The particle size of group Mix (3.05 μm) was nearly 2.6 times that of group 1% (1.18 μm). From these results, it can be speculated that pectin provides steric hindrance to the emulsion droplets, resulting in larger particle size but better emulsion stability. Similar research reported that dextran may enhance soy protein isolate steric hindrance leading to the better physical and structural stability of complex emulsion (28). Then, whether the complex formed by pectin and FG will affect the emulsification ability of FG will be analyzed in the subsequent interfacial properties analysis.

### Effect of Pectin and pH on the Properties of Complex Emulsions

#### Zeta Potential, Particle Size, and Dynamic Physical Stability of Complex Emulsions

Zeta potential and particle size are considerable indicators to evaluate the stability of an emulsion system. The zeta potential of each emulsion is shown in Figure 1D. The pI of FG was about pH 9; thus, the FE emulsion (pH 5.9) showed a positive charge. With the addition of pectin, the zeta potential transformed from a positive charge (18.97 mV) to a negative charge (−44.96 mV). Because the chains of pectin carry abundant -COOH (12), the zeta potential of complex emulsions became negative. The absolute value was much higher than that of the control FE. A previous study revealed that the high absolute zeta potential enhances emulsion stability (29). Van der Waals force and electrostatic interaction force in colloid constitute the main interaction between the two particles (30). Electrostatic repulsion in FG-pectin emulsion was dominant. FG-pectin emulsions were prone to more negative charges with the intensified alkalinity of solutions. However, the differences in zeta potentials at pH 6, 8, and 10 emulsions were not significant (*p* > 0.05). Because most -COOH in the FG or pectin chains transformed to -COO^–^, the negative charges were saturated. After emulsions were centrifuged (Supplementary Figure 2), the bottom layer of FG-pectin emulsions (pH < 6) were cloudy and the others were transparent. Gelatin and pectin can form coacervates *via* electrostatic interaction (31), so the cloudy of bottom layer was FG-pectin coacervates. With the increase of pH, the electrostatic interaction between FG and pectin weakened, making it difficult to form coacervates. The acid adjustment enhanced the zeta potential of emulsions, which were similar to 1.0% (*p* > 0.05).

The volume average particle size of emulsions is shown in Figure 1E. The particle size of emulsions increased first and then decreased as the pectin concentration increased. The particle size of emulsions containing 1 or 1.5% pectin was similar to that of FG emulsion (*p* > 0.05). High pectin content provided sufficient negative charge to enhance electrostatic and spatial repulsion. Similarly, previous research demonstrated that a large electrostatic repulsion force is exerted between the neighboring carrageenan (anionic polysaccharide) chains, and the carrageenan forms a much smaller complex with lentil protein in the solution (32). As solution alkalinity increased, the emulsion of pH 6 to 10 had a larger particle size from 3.85 to 7.51 μm. By comparison, pH 8 had the highest D_43_ (*p* < 0.05) possibly because it was close to the pI of FG. Under alkaline conditions, the electrostatic interactions between pectin and FG weakened, and the interactions of FG-pectin were dominated by surface patch binding (33). The acid adjustment group had a decreased particle size similar to 1.0% (*p* > 0.05).

Although the FG emulsion had the lowest particle size, the instability index of the FG emulsion was higher than that of FG-pectin emulsions (Figure 1F). Consistent with the zeta potential, the results showed that the stability of FG emulsion strengthened as pectin was mixed. The difference in DPS in the 0.5 to 1.5% pectin group was not significant (*p* > 0.05) during 30 min of centrifugation. In the comparison of the effect of pH on the DPS of emulsions, emulsions at pH 6 to 10 had the highest instability index, which indicated that the alkali-adjusted FG-pectin emulsions were unstable. This finding might be due to the weakened positive charge of FG and the larger particle size of the emulsion as in the previous analysis. During acid adjustment, DPS decreased evidently (*p* < 0.05). The results of zeta potential, particle size, and DPS indicated that the pH adjustment of the composite emulsion was reversible.

#### Microstructure of Complex Emulsions

**FIGURE 2:**
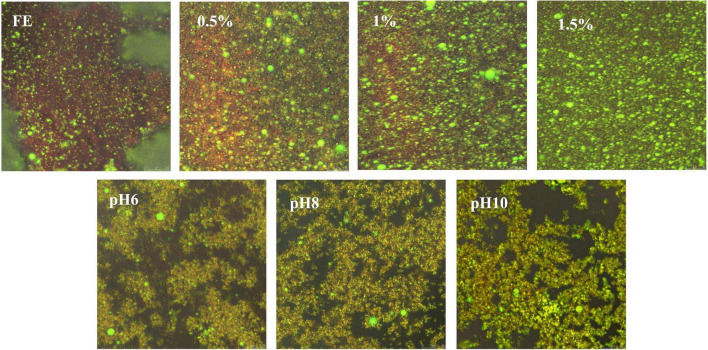
Confocal laser scanning microscopy of complex emulsions.

After being dyed, all fresh emulsions were immediately observed through CLSM (Figure 2). Nile blue was used to label the protein–polysaccharide content with green color intensity during the CLSM observation, and Nile red was utilized to label the internal oil phase (19). Amphiphilic substances converged at the oil–water interface and wrapped the oil droplets to form a sphere to prevent their aggregation. The oil droplets in the CLSM images were spherical, indicating that the oil droplet was successfully wrapped by the emulsifier. However, large accumulations were observed in the FG emulsion, demonstrating that FG molecules were prone to flocculation. Flocculation is a ubiquitous phenomenon that affects the emulsion, appearance, and rheology of O/W emulsions stabilized by proteins (34). On the contrary, FG emulsion mixed with pectin led to the disappearance of aggregates and a large number of loaded oil droplets. More loaded oil droplets formed because of the increase in the pectin content. When pH ≥ 6, clusters existed and the number of oil droplets decreased. The results showed that the inhomogeneity of alkali emulsions increased.

#### Storage Test of Complex Emulsions

**FIGURE 3:**
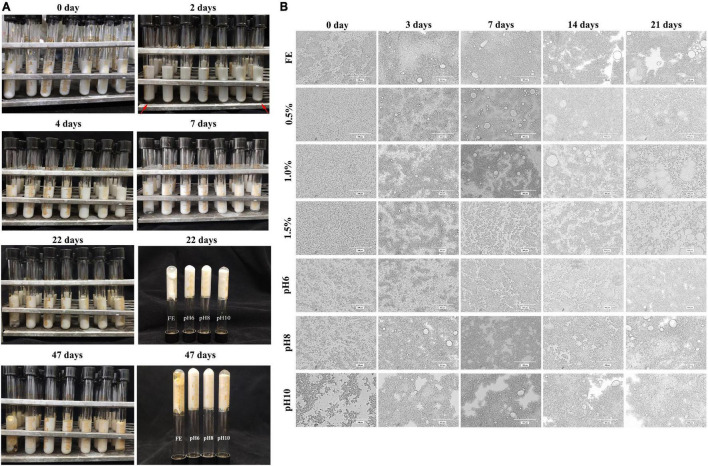
Storage measurement **(A)** and optical microscope observations **(B)** of complex emulsions.

During storage, unstable emulsions are prone to phase separation, flocculation, oil creaming, and precipitation (18). The storage stability of emulsions was determined during long-term storage at 25°C. The fresh emulsions of all the groups were white and fluid (Figure 3A). The FG emulsion was slightly stratified after 2 days of storage. With the extension of storage time, stratification became more serious. The emulsion at pH 10 was also stratified after 2 d of storage, which was slighter than the FG emulsion. In particular, the four emulsions (FG, pH 6 to 10) solidified after 22 d of storage. Consistent with our findings, previous results showed that gelatin emulsions undergo liquid–gel transition after 28 days of storage (10). By contrast, 0.5, 1.0, and 1.5% emulsions were not stratified and remained fluid during the whole storage. According to our previous research, pectin hinders the formation of FG gel networks (6), resulting in the loss of liquid–gel transition.

Optical microscope observations were performed to analyze the changes in the microstructure of emulsions during storage (Figure 3B). The oil droplets of fresh emulsions were small and non-conspicuous. Fresh FG emulsions flocculated to groups and FG-pectin emulsions (pH 6–10) were clustered, which were consistent with CLSM results. Conversely, FG-pectin emulsions (0.5, 1.0, and 1.5%) were uniform because polysaccharides can inhibit droplet movement (35). Combining with polysaccharides is an effective strategy to control and inhibit the flocculation of protein emulsions (34). As the storage time was extended, large oil droplets increased, implying that the droplets coalesced during this test. If oil droplets were not fully covered by particles, they tended to combine, thereby forming larger oil droplets (36). Interestingly, the clusters of FG-pectin emulsions (pH 6–10) assembled in groups in common with the observations of the FG emulsion. Thus, FG-pectin emulsions (pH 6–10) and FG emulsions were gelled in storage.

### Environmental Stability of Complex Emulsions

**FIGURE 4:**
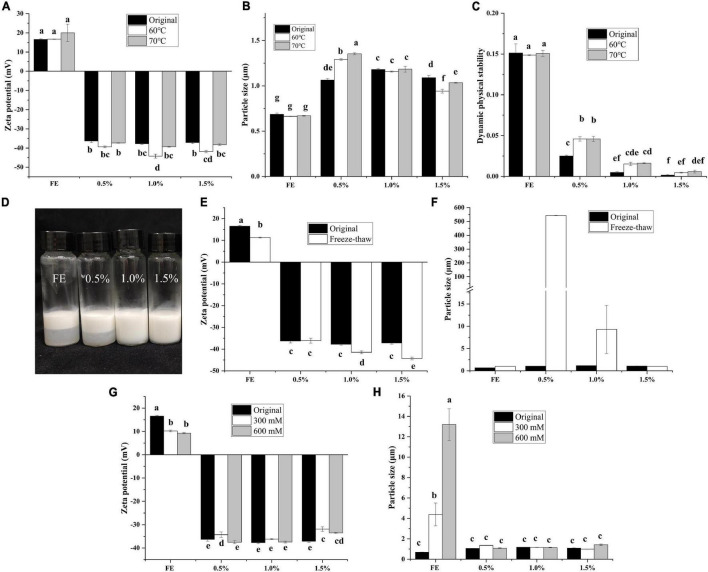
Effect of heat treatment on zeta potential **(A)**, particle size **(B)**, and dynamic physical stability **(C)** of complex emulsions; effect of freeze-thaw treatment on appearance **(D)**, zeta potential **(E)**, and particle size **(F)** of the complex emulsion; effect of freeze-thaw treatment on zeta potential **(G)** and particle size **(H)** of complex emulsion.

When emulsions are used in beverage or drug delivery, complex environments (high temperature, freeze-thaw, high salt) are often encountered, and the environmental stability of emulsions is indispensable at this time. The influence of thermal treatment on FG-pectin emulsions is shown in Figures 4A–C. The zeta potential, particle size, and DPS results indicated that FG emulsion was thermally stable. Although the high-temperature treatment weakened the stability of complex emulsions, the DPS of complex emulsions was still better than that of FG emulsion. All emulsions except 1.0 and 1.5% emulsions stratified after the freeze-thaw treatment (Figure 4D). After the freeze-thaw treatment, FG emulsions had a decreased zeta potential and enhanced particle size (Figures 4E,F). Most notably, abundant floccules appeared at 0.5% FG-pectin emulsions after the freeze-thaw treatment. Moreover, the particle size was larger than that of other groups, whereas the zeta potential did not change appreciably. Because of phase separation, polysaccharides and proteins combine to form insoluble agglomerates (14). When the emulsions were dominated by pectin, particle size and zeta potential decreased. This result demonstrated that the higher proportion of pectin in a composite emulsion was more conducive to the effect of freezing and thawing.

The influence of salt treatment on FG-pectin emulsions is shown in Figures 4G,H. FG emulsions were susceptible to salt concentration. The zeta potential decreased, and the particle size increased sharply. On the contrary, the complex emulsions were insensitive to changes in environmental salt concentrations. Therefore, FG-pectin emulsions were stable at high temperatures and could tolerate salt environments. When the pectin content in the composite emulsion was low, phase separation was likely to occur during the freeze-thaw process, but increasing the pectin content could avoid phase separation.

### Interaction Force Analysis

**FIGURE 5:**
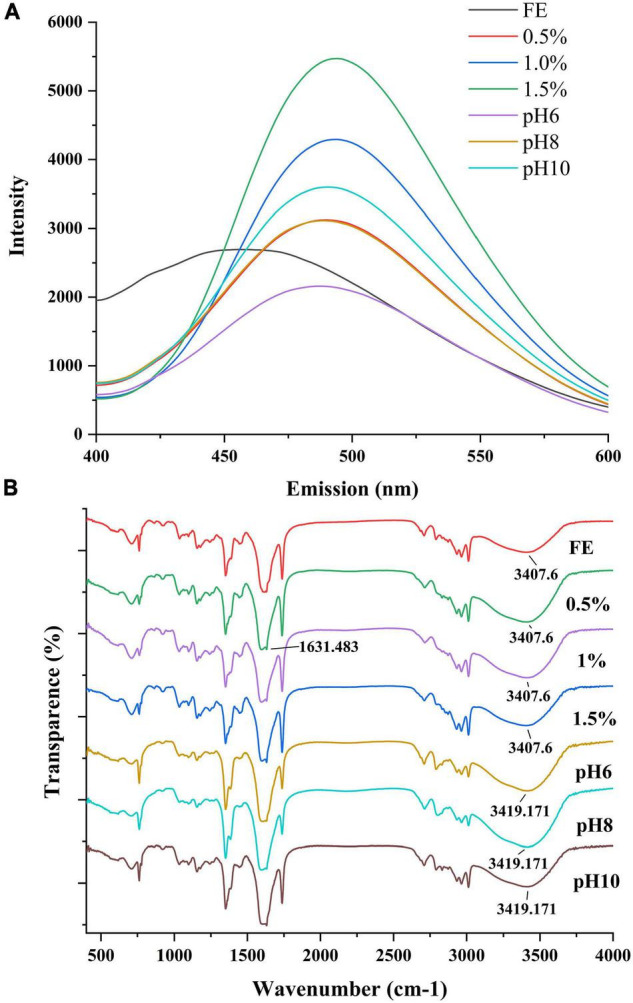
Surface hydrophobicity **(A)** and FTIR **(B)** of complex emulsions.

Hydrophobicity is related to the stability and functionality of proteins because of its effect on the conformation of proteins in solutions. The surface hydrophobicity of emulsions is shown in Figure 5A. FG emulsion had a peak at 456 nm with fluorescence intensity of 2,694. However, the fluorescence peaks were red-shift after mixing with pectin, and the fluorescence intensity improved as the pectin concentration increased. Therefore, the hydrophobic interaction of complex was promoted. This result was consistent with previous findings, which indicated that more hydrophobic groups of proteins are exposed because of the addition of polysaccharides (9). When the solution pH became neutral, the fluorescence intensity decreased because of the attenuation of FG-pectin binding. Then, as the solution became alkaline, the fluorescence intensity increased again. Under alkaline conditions, the FG chains aggregated because of the strengthened hydrophobic interaction (37).

FTIR was conducted to further detect the interaction force of the effect of pectin on FG emulsions (Figure 5B). The wide peak at around 3,407 cm^–1^ presented the N–H stretching and stretching vibration of hydroxyl groups, which are characteristic of amide A (38, 39). This peak did not change with the increase of pectin. However, in the alkali-adjusted groups, the peak at 3,407 cm^–1^ moved to 3,418 to 3,419 cm^–1^, which indicated that hydrogen bonds weakened. The peaks at 1,631 cm^–1^ belonged to amide I corresponded to C = O stretching or hydrogen bonds coupled with COO^–^ (9). Amide I in all the emulsions had no significant changes. Because of the stronger amide I absorption, the peaks at 1,616 cm^–1^ (1° amide II) were obscured by amide I. The 1° amide II corresponded to CN stretching and NH bending (38), which were moved to a lower wavenumber as the pectin concentrations increased. Conversely, these peaks at amide II initially moved to a higher wavenumber (pH < pI) and then moved to lower wavenumber (pH > pI) in the alkaline-adjusted emulsions.

The secondary structure of FG in emulsions was analyzed using the second derivative of the FTIR spectra in the amide I region (1,700–1,600 cm^–1^). The peaks corresponded to β-sheet (1,610–1,642 cm^–1^), random coil (1,642–1,650 cm^–1^), α-helix (1,650–1,660 cm^–1^), β-turn (1,660–1,680 cm^–1^), and anti-parallel β-sheets (1,680–1,700 cm^–1^) conformations (40). In Supplementary Figure 3, the secondary structures of FG in all emulsions were dominated by β-sheet, which was similar to a previous report (38). In addition, α-helix and random coil were reduced as pectin concentration increased to 1.0%; furthermore, the β-sheet substantially increased. This observation may be caused by the transformation from α-helix/random coil to β-sheet and the interaction between protein–lipid (41) and protein–polysaccharide molecules. The enhancement of the ordered structure indicated the higher stability of FG molecules. Phoon et al. (42) believed that the transition from a loose coil/α-helix to an intermolecular β-sheet is the key to establishing an effective interface barrier between oil and water. In the pH-adjusted groups, the β-sheet content decreased, but the random coil content initially increased and then decreased as pH increased. The growing tendency among the unordered secondary structures of FG displayed the increased openness and flexibility of protein molecules (42). It might also result in the flocculation of emulsions (pH 6–10).

### Rheology Properties of Complex Emulsion Analysis

**FIGURE 6:**
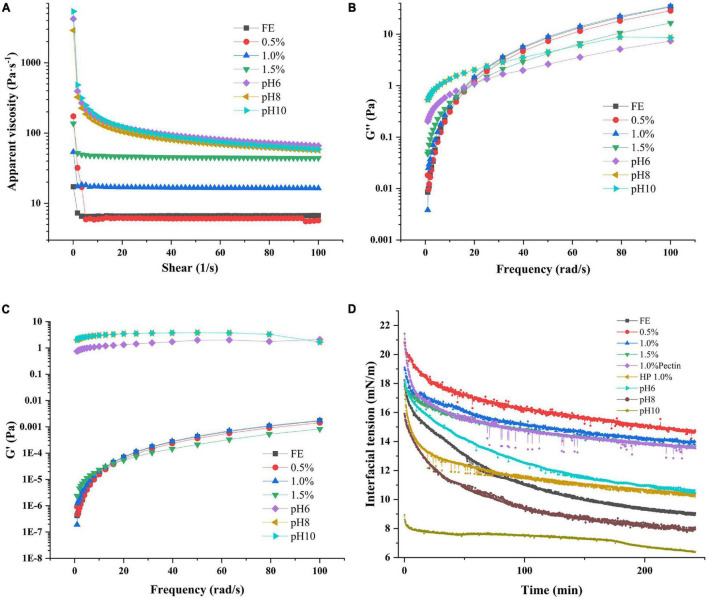
Apparent viscosity **(A)**, loss modulus **(B)**, storage modulus **(C)**, and interfacial tension **(D)** of complex emulsions.

The flow sweep test revealed the AV and fluid properties (Newtonian or non-Newtonian fluid) of emulsions. A Newtonian fluid is any fluid whose viscosity remains constant regardless of any external stress applied to it. On the contrary, the viscosity of a non-Newtonian fluid can change under force (6). As shown in Figure 6A, FG and FG-pectin emulsions (pH < 6) from 1 1/s to 100 1/s had constant AV, and the low pectin content of complex emulsions had a similar AV to that of the FG emulsion. High pectin concentrations positively affected the AV of emulsions, consistent with previous research that the viscosity of the polysaccharides-protein emulsion increased linearly with the polysaccharides concentration (43). Polysaccharides can strengthen the viscosity of emulsions and improve emulsion stability because of the extended network formation in the aqueous phase (44). Nevertheless, the AV of the emulsion at pH 6 to 10 groups was the highest, and it declined as the shear rate increased, displaying the features of a non-Newtonian fluid. This finding was due to the high flocculation of emulsion droplets during microstructure morphology observation. Compared with the nonflocculated FG-pectin emulsion, the flocculating emulsion showed an enhanced viscosity and shear-thinning behavior because some of the continuous phases were retained in the structure. These flocculates were destroyed by an external force, resulting in a shear-thinning behavior (29). As such, alkali-adjusted emulsions had a higher instability index but were only slightly stratified during storage.

Dynamic frequency sweep revealed the elasticity and viscosity of emulsions (Figures 6B,C). G′ (storage modulus) represents the elastic properties of emulsions and G″ (loss modulus) denotes the viscous properties of emulsions. The G″ of FE and FG-pectin emulsion (0.5–1.5%) under dynamic frequency scanning were much greater than that of G′, denoting that the viscous trend in the structure was dominant. Otherwise, alkali-adjusted emulsions had the highest G′ under low-frequency conditions, and G′ > G″ because of a high flocculation degree (45). With the increased frequency, G″ of alkali-adjusted emulsions approached G′, which was consistent with the flow sweep test results, that is, flocculation dispersed.

### Interfacial Properties Analysis

The interaction between polysaccharide and protein is affected by the pH value of the solution, the ratio of biopolymers, the order of adding biopolymers, and other factors (46), and these effects may further affect the surface activity of biopolymers. The change in the surface pressure of FG and pectin adsorbed on the oil–water interface with adsorption time was analyzed and detected *via* dynamic drop analysis. The interfacial tension of FG and FG-pectin is presented in Figure 6D. All interfacial tension–time curves are divided into rapid and slow reduction stages. For the FG emulsion, the interfacial tension decreased to 9.05 (mM/m) near the steady phase, indicating a good surface activity. However, the same concentration of pectin decreased to 13.53 (mM/m) near the steady phase, showing a poor surface activity because of the lack of a hydrophobic unit (12), which limited their adsorption at the oil–water interface. After FG was mixed with pectin *via* stirring and shearing, the interfacial tension increased. As the pectin content increased, the curves were similar to those of pectin. When the amount of pectin was enough, the interfacial tension slightly changed. This result is similar to that reported by Wang et al. (32), who demonstrated that the interaction between a lentil protein isolate and an anionic polysaccharide binding complex may limit the rearrangement of correct protein localization at the interface; as a result, interfacial tension increases. The interfacial tension of the complex at pH 6-10 were decreased due to the formation FG-pectin coacervate were prevented. In the case of pH 8/10, the interfacial tension of the composite emulsion is below the pure FG emulsion. These results show that an alkaline environment is more conducive to FG molecules’ adsorption. The alkaline environment contributes to the interfacial properties of gelatin (47). Thus, the higher the solution pH, the lower the interfacial tension value.

Interfacial property analysis consequence of emulsifiers revealed that the pectin and FG mixing had an antagonistic effect on the adsorption of oil–water interface. This antagonism effect may be owing to the adsorption of pectin at the interface to prevent FG molecules from approaching or the decrease of adsorption capacity of the FG-pectin coacervate. To further explore this question, the high-pressure homogenization group was designed. HP 1.0% group had a lower interfacial tension than the mixing 1.0%. During homogenization, pectin chains and proteins are rapidly activated and strengthened under shear force (14). High-pressure homogenization deepened the interaction between FG and pectin and improved the surface activity of the composite.

### Schematic Model of Fish Gelatin-Pectin Complex Emulsion Interactions

**FIGURE 7:**
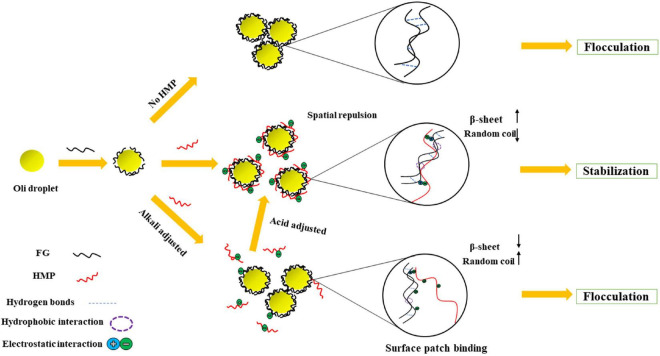
Schematic mechanism of FG-pectin complex emulsions.

The proposed potential mechanism of FG-pectin emulsion interactions is illustrated in Figure 7. In the presence of amphiphilic macromolecular particles at high concentrations, they can quickly adsorb to the oil surface and prevent their coalescence through space and electrostatic repulsion (36). According to the theory of bridging (48), FG emulsion could easily flocculate because of the intermolecular hydrogen bonds effect, leading to emulsion stratification and liquid–gel transition behavior. When pH < 6, the AV of the FG-pectin emulsion was higher than that of the FE emulsion. When pectin was added, the β-sheet of the complex increased and the random coil decreased. FG emulsion flocculation was prevented by increasing the hydrophobic interaction and electrostatic interaction of the FG-pectin system, ultimately enhancing the emulsion stability. The salinity tolerance of complex emulsions was improved by pectin. But the low-pectin-content emulsions underwent phase separation to form an insoluble complex during the freeze-thaw treatment. Hence, complex emulsions should contain a high amount of pectin, which was more stable in freeze-thaw and heat treatment processes. When pH ≥ 6, the emulsion was a non-Newtonian fluid whose AV and G′ were higher than those of emulsions at pH < 6. Emulsion clusters with a large particle size formed. During long-term storage, the emulsions were prone to stratification and gelling but were still lighter than the FE emulsion. Although the total potential decreased, the electrostatic barrier between particles was destroyed. The electrostatic interaction of FG-pectin shifted to surface patch binding, whereas the hydrophobic interaction and hydrogen bonds were decreased. Therefore, FG and pectin formed a compatible phase system, resulting in emulsion flocculation. However, this process could be reversed by acid adjustment and homogenization, making it simpler and more convenient in practical applications.

## Conclusion

Pectin greatly prolonged the storage property of fish gelatin emulsion, prevented delamination and gelation during storage, and enhanced the salt ion tolerance of FG emulsion. The fish gelatin emulsion was easy to delaminate during the freeze-thaw process, and the high pectin content improved the freeze-thaw stability of the fish gelatin emulsion. Since both FG and pectin are biological macromolecules, antagonism occurred during the adsorption process on the surface of oil droplets, increasing surface tension of the complex. High-pressure homogenization was required to allow sufficient interaction of FG and pectin, thereby enhancing the surface activity of the complex. The pH had a great influence on the complex emulsion, and the emulsion was unstable when pH ≥ 6. However, the stability of FG-pectin emulsion induced by pH adjustment was reversible due to the non-covalent interactions of FG-pectin. Pectin could make up for the corresponding defects of FG in emulsifying properties and form stable emulsions. This study showed great potential for nutrient or drug delivery and needed further studies.

## Data Availability Statement

The original contributions presented in this study are included in the article/Supplementary Material, further inquiries can be directed to the corresponding author.

## Author Contributions

SH carried out the major experiments and data analysis and writing – original draft preparation. ZT contributed to the supervision, funding, and review the manuscript. HW carried out the part of the experiments and analyzed the relative data. XS reviewed the manuscript. SW contributed to the software and validation. NC assisted to carry out the experiments. YH reviewed the manuscript. All authors contributed to the article and approved the submitted version.

## Conflict of Interest

The authors declare that the research was conducted in the absence of any commercial or financial relationships that could be construed as a potential conflict of interest.

## Publisher’s Note

All claims expressed in this article are solely those of the authors and do not necessarily represent those of their affiliated organizations, or those of the publisher, the editors and the reviewers. Any product that may be evaluated in this article, or claim that may be made by its manufacturer, is not guaranteed or endorsed by the publisher.
