# Supplementary material for: Pectin Stabilized Fish Gelatin Emulsions: Physical Stability, Rheological, and Interaction Properties
Source: Front Nutr. 2022 Jul 13;9:961875. doi: 10.3389/fnut.2022.961875 (PMC9326445; doi:10.3389/fnut.2022.961875)
Supplement: Supplementary file 1 [file Data_Sheet_1.PDF]

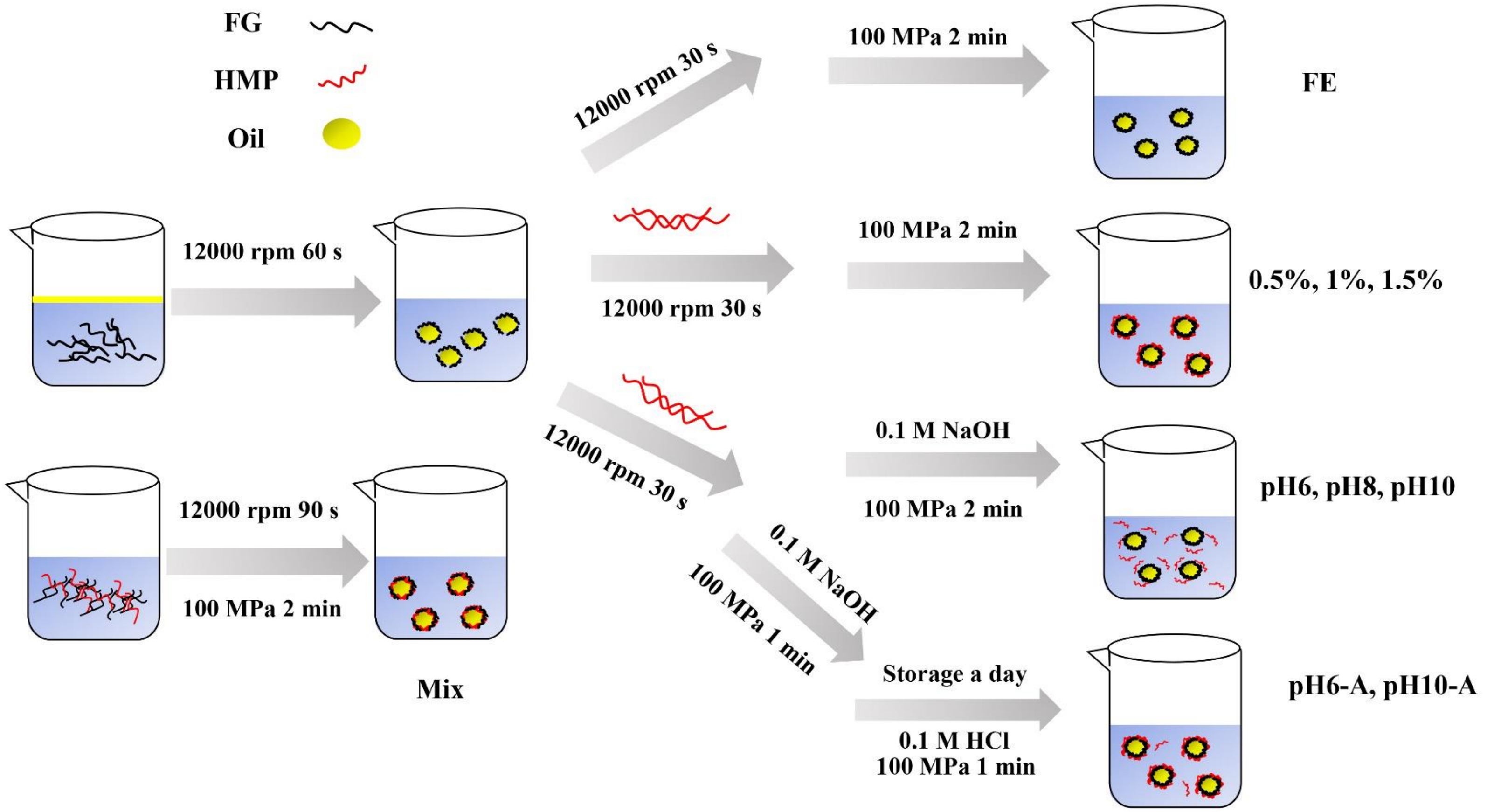

Fig. S1 Schematic diagram of the emulsion preparation process

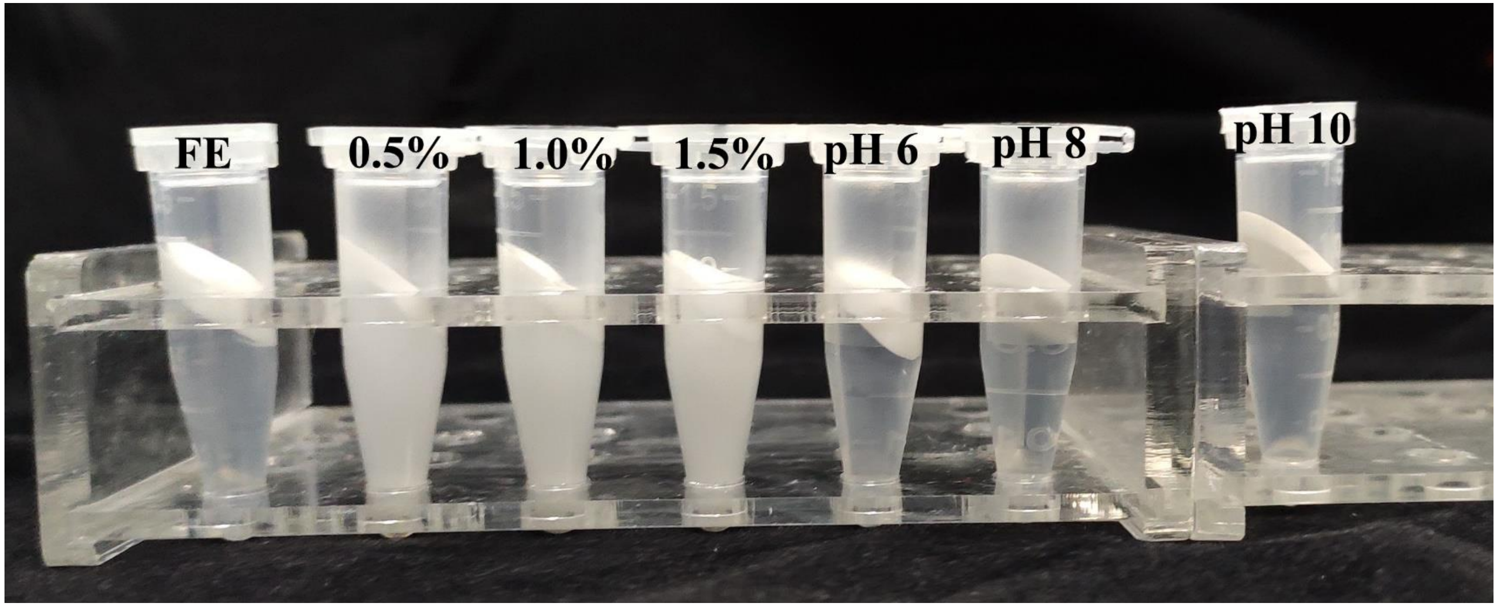

Fig. S2 Complex emulsions after centrifugation

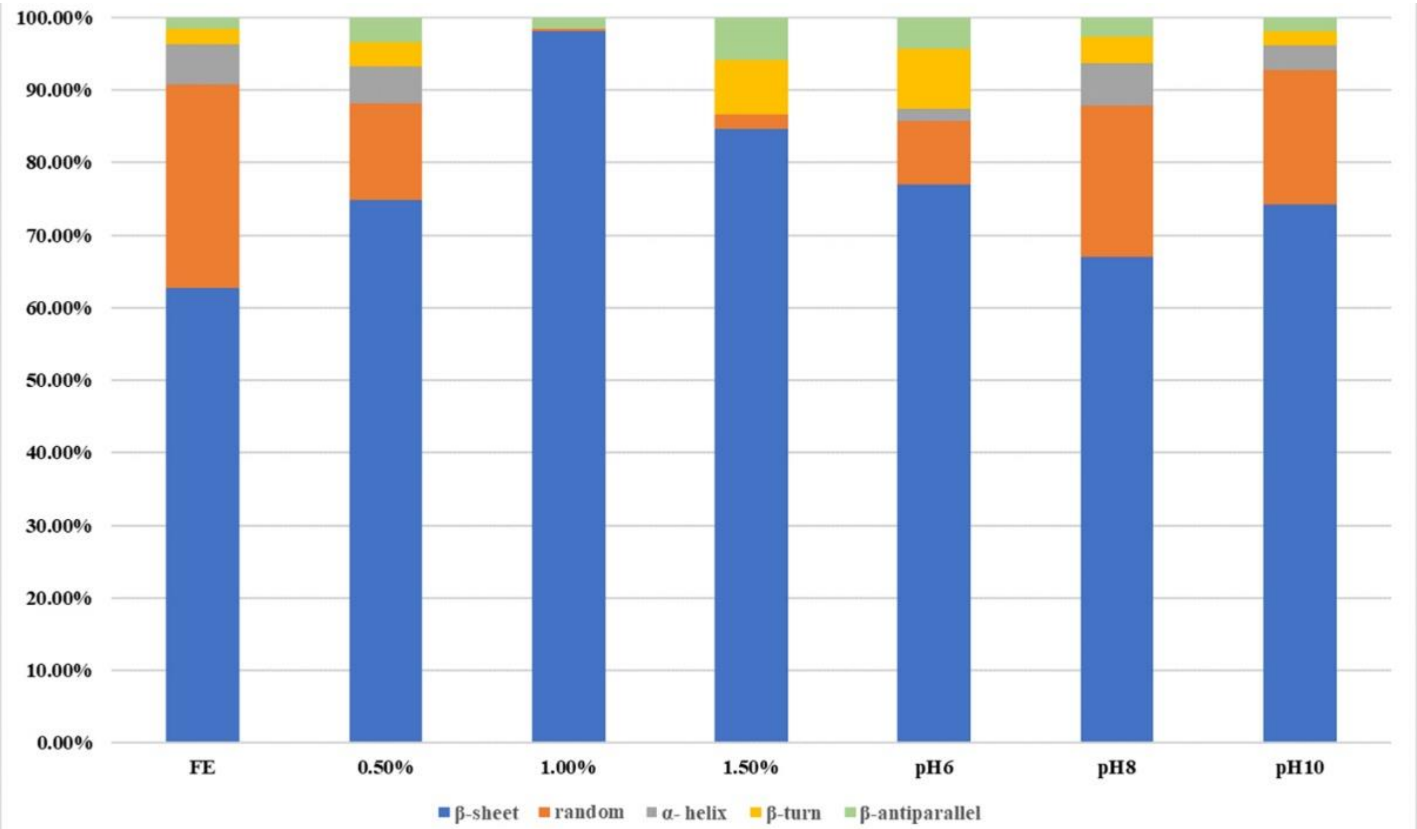

Fig. S3 Secondary structure of protein in complex emulsions
